# Incidence, Etiology and Outcomes of Hyponatremia after Transsphenoidal Surgery: Experience with 344 Consecutive Patients at a Single Tertiary Center

**DOI:** 10.3390/jcm3041199

**Published:** 2014-10-28

**Authors:** Sean M. Barber, Brandon D. Liebelt, David S. Baskin

**Affiliations:** Department of Neurosurgery, Houston Methodist Neurological Institute and the Kenneth R, Peak Brain and Pituitary Tumor Treatment Center, 6560 Fannin St. Suite 944, Houston, TX 77030, USA

**Keywords:** hyponatremia, SIADH, pituitary adenoma, transsphenoidal, cerebral salt wasting

## Abstract

Hyponatremia is often seen after transsphenoidal surgery and is a source of considerable economic burden and patient-related morbidity and mortality. We performed a retrospective review of 344 patients who underwent transsphenoidal surgery at our institution between 2006 and 2012. Postoperative hyponatremia was seen in 18.0% of patients at a mean of 3.9 days postoperatively. Hyponatremia was most commonly mild (51.6%) and clinically asymptomatic (93.8%). SIADH was the primary cause of hyponatremia in the majority of cases (*n* = 44, 71.0%), followed by cerebral salt wasting (*n* = 15, 24.2%) and desmopressin over-administration (*n* = 3, 4.8%). The incidence of postoperative hyponatremia was significantly higher in patients with cardiac, renal and/or thyroid disease (*p* = 0.0034, Objective Risk (*OR*) = 2.60) and in female patients (*p* = 0.011, *OR* = 2.18) or patients undergoing post-operative cerebrospinal fluid drainage (*p* = 0.0006). Treatment with hypertonic saline (*OR* = −2.4, *p* = 0.10) and sodium chloride tablets (*OR* = −1.57, *p* = 0.45) was associated with a non-significant trend toward faster resolution of hyponatremia. The use of fluid restriction and diuretics should be de-emphasized in the treatment of post-transsphenoidal hyponatremia, as they have not been shown to significantly alter the time-course to the restoration of sodium balance.

## 1. Introduction

Hyponatremia is a common finding in both the neurosurgical patient population and the inpatient population in general [1,2]. Subarachnoid hemorrhage [3,4,5,6], traumatic brain injury [7,8,9,10], spinal cord injury [11,12], meningitis [13,14,15], neurosurgical procedures [16] and a multitude of other CNS-related pathologies have been shown to predispose neurosurgical patients to hyponatremia.

Hypernatremia related to diabetes insipidus (DI) is perhaps the most anticipated electrolyte abnormality to occur after transsphenoidal surgery, but hyponatremia has in fact been shown to occur with greater frequency and result in a greater degree of patient morbidity in some series [17,18,19,20,21]. Indeed, hyponatremia in general, whether adequately treated or not, is associated with a considerable degree of morbidity, mortality and healthcare-related economic burden (with an estimated annual cost of $3.6 billion) [1,22,23,24].

Although post-transsphenoidal hyponatremia is often attributed to syndrome of inappropriate antidiuretic hormone (SIADH), early evidence indicates that a variety of other etiologies may be implicated [19,24], though the precise etiology of hyponatremia in any given case is often difficult to assess accurately. Characterizations of clinical diagnostic criteria delineating SIADH from cerebral salt wasting (CSW) (e.g., volume status) are notoriously unreliable in the absence of invasive monitoring techniques (e.g., Swan-Ganz catheterization) [25,26,27,28], for example. Diagnostic criteria based on accurately quantifiable factors (e.g., plasma urea, atrial natriuretic peptide, antidiuretic hormone and other serum markers) have not been demonstrated to be accurate predictors of hyponatremia etiology in all cases, and their use in the evaluation of hyponatremia is not supported by the literature (Class III evidence) [24]. Further complicating matters is that the differential diagnosis of hyponatremia, even after transsphenoidal surgery, includes a number of possibilities (e.g., including secondary adrenal insufficiency, over-administration of desmopressin acetate and renal, cardiac or thyroid dysfunction, among others).

In the interest of improving the outcomes of patients with hyponatremia after transsphenoidal surgery, an accurate and thorough understanding of the incidence, etiology and outcomes of post-transsphenoidal hyponatremia is necessary. In this series, a large, retrospective review of patients undergoing transsphenoidal surgery at the authors’ institution is provided in the interest of better characterizing the incidence, etiology and outcomes of hyponatremia related to transsphenoidal surgery, while identifying patient and procedure-related factors associated with an elevated risk of post-transsphenoidal hyponatremia development.

## 2. Methods

The details of this study were reviewed and approved by the Houston Methodist Hospital Institutional Review Board, Protocol number 00011946. The study was conducted with high ethical standards in accordance with the IRB approved protocol. The authors retrospectively reviewed a database of patients treated at the authors’ institution between January, 2006, and December, 2012. Three-hundred forty four consecutive patients were admitted for transsphenoidal resection of a sellar and/or suprasellar mass at our institution by a single senior staff neurosurgeon during this time period (David S. Baskin). After obtaining approval from all 344 patients for the use of their medical records in retrospective analysis, data regarding patient demographics, clinical presentation, comorbidities, pituitary tumor morphology and histology, pre- and post-operative laboratory values, date and severity of hyponatremia, treatments employed for hyponatremia, rate of hyponatremia correction and clinical outcomes on clinical follow-up visits were collected for each patient.

A single routine pre-operative serum sodium level was drawn for all patients per institutional protocol, and daily post-operative metabolic panels were drawn while the patient remained within the Neurological Intensive Care Unit (NICU). Postoperative hyponatremia was defined as a serum sodium value less than 135 mEq/L occurring within 30 days of surgery. Hyponatremia severity was classified according to serum sodium levels as mild (131–134 mEq/L), moderate (125–130 mEq/L) or severe (<125 mEq/L). Hyponatremia was not present immediately prior to surgery in any case.

The functional status of each pituitary adenoma was defined according to the results of immunohistochemistry stains for the given hormone performed on permanent sections of surgical specimens in combination with an elevated serum level of the given hormone. Pre- and post-operative tumor size was determined in each case by measurements taken from preoperative magnetic resonance images (MRIs) of the brain and reported as the largest single diameter of the tumor in any of the three orthogonal planes. Tumor volumes were estimated by approximating the tumor as a sphere with the largest tumor diameter being the diameter of the sphere.

The etiology of hyponatremia in each case was distinguished based on clinical and, in some cases, laboratory data, as described elsewhere [2,24,29,30]. Although serum osmolarity was infrequently measured (measured in 15 patients with hyponatremia, 24.2%), hyponatremia was presumed to be hypotonic in all other cases, as no evidence of severe hyperglycemia or recent administration of mannitol or sodium-free irrigant solutes was present. SIADH was defined as hypotonic hyponatremia in a euvolemic or hypervolemic patient without evidence of elevated cortisol, disturbance in thyroid function or excessive intake of hypotonic solution (e.g., polydipsia). CSW was defined as hypotonic hyponatremia in a hypovolemic patient without clinical evidence of diarrhea or excessive blood loss, vomiting, or sweating. Urine sodium was ordered as a confirmatory measure in 10 patients (16.1%) with hyponatremia.

Volume status was estimated for each patient at the time of hyponatremia based on strict intake/output volumes documented for each patient (hypovolemia was defined as a volume deficit >1 liter since admission; hypervolemia was defined as a volume excess >1 liter since admission; and euvolemia was defined as a volume excess or deficit ≤1 since admission), vital signs (where hypotension out of the proportion to baseline blood pressures or orthostatic vital signs changes were considered consistent with hypovolemia), physical exam findings and evaluation of chest plain films (where pulmonary edema or prominent vascular congestion was considered consistent with volume excess).

All statistical analyses were performed using SPSS 15.0 (SPSS Inc., Chicago, USA). The threshold for statistical significance was set at α = 0.05. Continuous data are presented as the mean ± SD; categorical and nominal data are presented as frequency and percentage. Univariate Fisher exact tests and Student’s *t*-tests were used to interrogate individual characteristics that might be associated with postoperative hyponatremia, and logistic regressions analysis was performed to assess the influence of multiple factors on outcome patterns.

## 3. Results

### 3.1. Patient Characteristics

The patient population consisted of 344 consecutive patients, including 153 men and 191 women (mean age, 48 ± 16 years; range, 15–84 years). The most common comorbidities included hypertension (144 patients, 41.9%), diabetes mellitus (56 patients, 16.3%), primary hypothyroidism (42 patients, 12.2%), hyperlipidemia (21 patients, 6.1%) and coronary artery disease (17 patients, 4.9%). All patients with hypothyroidism were well-controlled with medication prior to surgery. The median American Society of Anesthesiologists (ASA) score was two (range, 1–4; mean, 2.45 ± 0.57). Patient characteristics are summarized in Table 1.

**Table 1:** Age, sex and comorbidities of 344 consecutive patients who underwent transsphenoidal resection of a pituitary mass at the authors’ institution between January, 2006, and December, 2012. GERD = gastroesophageal reflux disease; PCOS = polycystic ovary syndrome; BPH = benign prostatic hyperplasia.

Number of patients	344
Mean age	48 ± 16 years
**Gender (*N* (%))**	
Male	153 (44.5)
Female	191 (55.5)
**Comorbidities (*N* (%))**	
Hypertension	127 (36.9)
Diabetes mellitus	56 (16.3)
Hypothyroidism	42 (12.2)
Hyperlipidemia	21 (6.1)
Coronary artery disease	17 (4.9)
Atrial fibrillation	4 (1.2)
GERD	4 (1.2)
Asthma	4 (1.2)
Cerebro-occlusive disease	4 (1.2)
Breast cancer	4 (1.2)
Depression	4 (1.2)
Anxiety	3 (0.9)
Migraines	3 (0.9)
Obstructive sleep apnea	3 (0.9)
PCOS	3 (0.9)
BPH	2 (0.6)
Renal cell carcinoma	2 (0.6)
Sickle cell trait	2 (0.6)
Rheumatoid arthritis	2 (0.6)

### 3.2. Tumor Characteristics

The most common intrasellar and/or suprasellar pathologies included null-cell adenoma (228 patients, 66.3%), functional adenoma (55 patients, 16.0%) and Rathke’s cleft cyst (48 patients, 14.0%). The most common subtypes of functional adenoma were prolactinoma (23 patients, 41.8% of functional adenomas) and somatotroph adenoma (10 patients, 18.2% of functional adenomas). The mean tumor size was 6.37 ± 10.4 cm^3^ preoperatively. Post-operative imaging was available in 286 of the 344 patients (83.1%), obtained at a mean of 2.2 months postoperatively. The mean postoperative tumor size in these 286 patients was 1.03 ± 4.55 cm^3^. Gross total resection was seen in 182 of these 286 patients (63.6%). The mean absolute decrease in tumor size after surgery in these 286 patients was 5.75 cm^3^, whereas the mean relative decrease in tumor size in these 286 patients was 90.7%. Tumor characteristics are summarized in Table 2.

**Table 2:** Tumor size, resection success and anatomic pathology for 344 consecutive patients who underwent transsphenoidal resection of a pituitary mass at the authors’ institution between January, 2006, and December, 2012. PRL = prolactin; GH = growth hormone; LH = luteinizing hormone; FSH = follicle stimulating hormone; ACTH = adrenocorticotropic hormone.

Mean Tumor Size	
Preoperative (*N* = 344)	6.37 ± 10.4 cm^3^
Postoperative (*N* = 286)	1.03 ± 4.55 cm^3^
**Completeness of Resection**	
Gross total resection (*N* (%))	182 (63.6)
Mean absolute tumor resection (cm^3^)	5.75
Mean relative tumor resection (%)	90.7
**Tumor Pathologies (*N* (%))**	
Null cell adenoma	228 (66.3)
Functional adenoma	55 (16.0)
Prolactinoma (PRL)	23 (6.8)
Somatotroph adenoma (GH)	10 (2.9)
LH/FSH	5 (1.5)
LH	4 (1.2)
Corticotroph adenoma (ACTH)	4 (1.2)
FSH	2 (0.6)
PRL/FSH	1 (0.3)
PRL/LH	1 (0.3)
PRL/ACTH	1 (0.3)
PRL/ACTH/GH	1 (0.3)
ACTH/FSH	1 (0.3)
ACTH/GH	1 (0.3)
ACTH/LH/FSH	1 (0.3)
Rathke’s cleft cyst	48 (14.0)
Chordoma	3 (0.9)
Lymphocytic adenohypophysitis	2 (0.6)
Null cell adenoma + Rathke’s cleft cyst	2 (0.6)
Somatotroph adenoma (GH) + Rathke’s cleft cyst	1 (0.3)
Craniopharyngioma	1 (0.3)
Germinoma	1 (0.3)
Chondrosarcoma	1 (0.3)
Fibrous dysplasia	1 (0.3)

### 3.3. Preoperative Anterior Pituitary Function

Preoperative endocrine laboratory values were available in 285 of the 344 patients (82.8%). Preoperative hypopituitarism was evident in 130 of these 285 patients (45.6%). The most common pre-operative deficits in hormones of the anterior pituitary included: testosterone (80 patients, 61.5% of those with preoperative hypopituitarism), GH (growth hormone)/IGF-1 (36 patients, 27.7% of those with preoperative hypopituitarism), ACTH (adrenocorticotropic hormone) (28 patients, 21.5% of those with preoperative hypopituitarism) and TSH (28 patients, 21.5% of those with preoperative hypopituitarism).

### 3.4. Surgical Procedure

All procedures were performed with the patient in the semi-sitting position under general anesthesia. Preoperative antibiotics were given in all cases and included vancomycin and cefepime, except in the case of documented allergy to either agent. A lumbar drain was placed preoperatively in all patients with suprasellar tumor extension. In all 344 patients, the tumor was approached and resected through a transnasal, transsphenoidal route after the administration of topical cocaine and oxymetazoline to the nasal mucosa. An endoscope was typically used for the initial approach and resection, followed by further resection with the surgical microscope. In the case of some tumors with suprasellar extension, visualization and/or resection of the mass was aided by the infusion of saline into the lumbar drain, resulting in a pressure-induced migration of tumor material inferiorly from the suprasellar space into the operative field. After resection was complete, closure frequently involved placement of an abdominal fat graft and a porous polyethylene implant (Medpor^©^, Porex Surgical, Inc., Newnan, GA, USA) with or without overlying dural sealant (DuraSeal^®^, Covidien, Waltham, MA, USA).

### 3.5. Hospital Course

After anesthesia was discontinued, the patient was extubated and awakened in the operating room before being transported to a recovery room. All patients were placed in the NICU for at least one night and were later transferred to an acute care floor for further convalescence prior to discharge. The mean ICU stay was 2.7 ± 2.6 days (range, 1–16 days) for the 344 study patients, and the mean total hospital stay was 4 ± 3.3 days (range, 1–21 days). The development of post-operative hyponatremia of any severity or etiology was a significant predictor of both prolonged hospital stay (*OR* = 2.82, *p* < 0.0005) and prolonged ICU stay (*OR* = 1.43, *p* < 0.0005).

### 3.6. Incidence of Postoperative Hyponatremia

**Figure 1:**
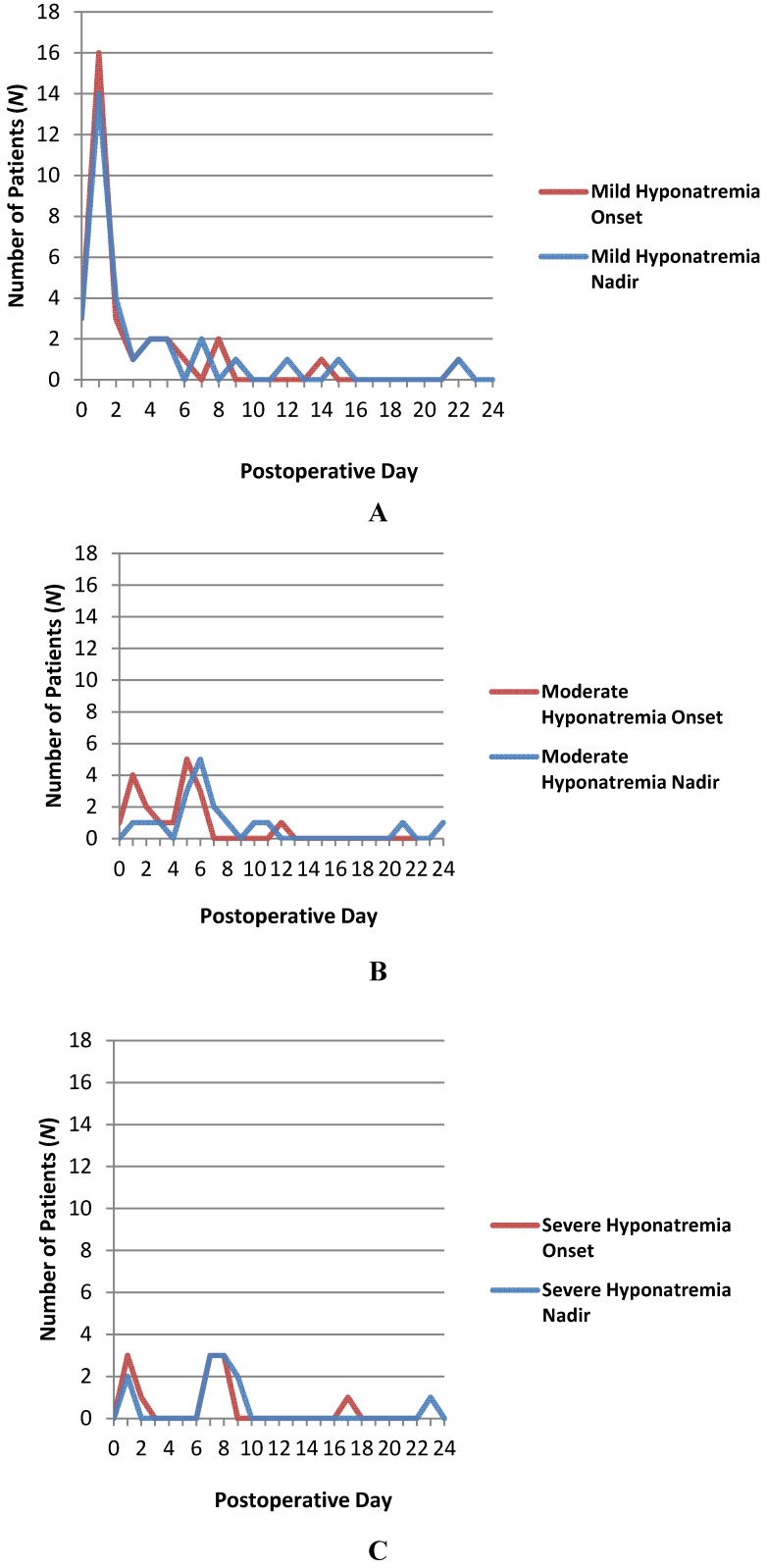
Line graphs illustrating the day of hyponatremia onset (the first documented serum sodium <135 mEq/L) and nadir in each of the three severity groups (**A**: mild; **B**: moderate; **C**: severe hyponatremia) in 62 patients with post-operative hyponatremia in a group of 344 consecutive patients who underwent transsphenoidal resection of a pituitary mass at the authors’ institution between January, 2006, and December, 2012.

Postoperative hyponatremia was seen in 62 of the 344 patients (18.0%) at a mean of 3.9 ± 4.2 days postoperatively. Mild hyponatremia (serum sodium 131–134 mEq/L) was seen in the majority of these cases (32 patients, 51.6% of those with postoperative hyponatremia), followed in frequency by moderate hyponatremia (serum sodium 125–130 mEq/L, 19 patients, 30.6% of those with postoperative hyponatremia) and severe hyponatremia (serum sodium <125 mEq/L, 11 patients, 17.7% of those with postoperative hyponatremia). The onset of hyponatremia (*i.e.*, the first documented serum sodium <135 mEq/L) occurred significantly earlier in the postoperative course for mild hyponatremia (mean time of onset, 3.2 ± 4.6 days postoperatively) when compared with severe hyponatremia (mean time of onset, 6.1 ± 4.8 days postoperatively; *p* = 0.038). Moderate hyponatremia frequently arose at a time point between mild and severe hyponatremia (mean time of onset, 4.0 ± 2.8 days postoperatively). The nadir of hyponatremia (day of lowest serum sodium concentration) occurred significantly earlier in the postoperative course for mild hyponatremia (mean day of nadir, 3.6 ± 4.9 days postoperatively) when compared with moderate (mean day of nadir, 7.7 ± 5.9 days postoperatively; *p* = 0.005) or severe hyponatremia (mean day of nadir, 8.0 ± 5.7 days postoperatively; *p* = 0.009). Trends in hyponatremia onset and nadir are illustrated in Figure 1.

The likelihood of developing hyponatremia was significantly greater in patients subjected to post-operative CSF diversion (47.2% risk of hyponatremia development) when compared with patients without lumbar drains or those in whom the drain remained clamped post-operatively (16.2% risk of hyponatremia development; *p* = 0.0006). Drainage of CSF through a lumbar drain occurred in 78 (22.7%) of study patients at a mean rate of 249 ± 62.6 cc/day over a mean of 4.4 ± 1.1 days. Neither the length (in days; *R* = 0.023, *p* = 0.91) of CSF drainage nor the rate (in mL/day; *R* = 0.11, *p* = 0.59) of CSF drainage correlated with the severity of hyponatremia in these 78 patients.

Hypernatremia was observed in the postoperative period in nine of the 62 patients (14.5%) with post-transsphenoidal hyponatremia. A classic triphasic post-transsphenoidal response (of diabetes insipidus, followed by SIADH, followed by diabetes insipidus) was observed in only a single patient (1.6% of patients with hyponatremia) who underwent transsphenoidal resection of a suprasellar germinoma, although the hyponatremia in this patient may have been partially related to overdosing of desmopressin acetate for DI. Two patients exhibited a biphasic response wherein DI was seen initially and was followed later by SIADH (3.2% of patients with hyponatremia), and two other patients exhibited a biphasic response wherein SIADH occurred initially and was followed by DI (3.2% of patients with hyponatremia).

### 3.7. Clinical Manifestations of Hyponatremia

The majority of patients with mild hyponatremia reported no clinical symptoms attributable to hyponatremia (93.8% asymptomatic), although a single patient complained of nausea, and another patient complained of a mild headache. Symptoms attributable to hyponatremia were seen in 36.8% (*n* = 19) of those with moderate hyponatremia and 90.9% (*n* = 11) of those with severe hyponatremia. In eight patients (12.9% of those with hyponatremia), clinical symptoms arose after discharge from the hospital postoperatively and prompted an evaluation in the clinic or the Emergency Room and subsequent readmission for treatment of hyponatremia (moderate, *n* = 4; severe, *n* = 4). A single patient was readmitted postoperatively for treatment of a cerebrospinal fluid (CSF) leak and incidentally found to have a serum sodium level of 125 mEq/L, requiring treatment, though he reported no symptoms attributable to hyponatremia. No seizures or coma occurred in any hyponatremic patient. Clinical manifestations of postoperative hyponatremia are summarized in Table 3.

**Table 3:** Incidence, clinical symptomatology and etiologies of postoperative hyponatremia in 344 consecutive patients who underwent transsphenoidal resection of a pituitary mass at the authors’ institution between January, 2006, and December, 2012.

Incidence of Post-operative Hyponatremia (*N* (%))	62 (18.0)
**Hyponatremia Severity (*N* (%))**
Mild (131–134 mEq/L)	32 (51.6)
Moderate (125–130 mEq/L)	19 (30.6)
Severe (≤124 mEq/L)	11 (17.7)
**Clinical Symptoms (*N* (%))**
None	43 (69.4)
Nausea/Vomiting	10 (16.1)
Headache	4 (6.5)
Malaise	3 (4.8)
Agitation	1 (1.6)
Confusion	1 (1.6)
Balance difficulty	1 (1.6)
Abdominal pain	1 (1.6)
**Primary Etiology (*N* (%))**
Syndrome of inappropriate ADH secretion (SIADH)	44 (71)
Cerebral salt wasting (CSW)	15 (24.2)
Desmopressin acetate overdose	3 (4.8)
**Secondary Etiology (*N* (%))**
Hypoadrenal state	4 (6.5)
Desmopressin acetate overdose	3 (4.8)
Ischemic cardiomyopathy	1 (1.6)
Excessive oral intake of hypotonic fluids	1 (1.6)

### 3.8. Etiology of Hyponatremia

Syndrome of inappropriate antidiuretic hormone secretion (SIADH) was determined to be the primary cause of hyponatremia in the large majority of cases (*n* = 44, 71.0%), followed in frequency by cerebral salt wasting (*n* = 15, 24.2%) and over-administration of desmopressin acetate (*n* = 3, 4.8%). SIADH was the predominant etiology for each of the three hyponatremia severity groups, both independently and as a whole, as defined by criteria described above. Severe hyponatremia (serum sodium <125 mEq/L), in particular, was almost exclusively due to SIADH (10/11 cases, 90.9%). In seven patients with hyponatremia determined to be due primarily to SIADH and a single patient with hyponatremia due primarily to CSW, other etiological factors, such as hypoadrenal state (*n* = 4), overuse of desmopressin acetate (*n* = 3), ischemic cardiomyopathy (*n* = 1) or excessive oral intake of hypotonic fluids (*n* = 1) were also felt to play a role in the development of hyponatremia. Multifactorial etiologies for hyponatremia were most common in the severe hyponatremia group, where six of the 11 patients (54.5%) were thought to have several different factors contributing to hyponatremia development. Etiologies of postoperative hyponatremia are summarized in Table 3.

Logistic regression analysis revealed that the likelihood of developing postoperative hyponatremia of any severity and due to any etiology was significantly higher in patients with cardiac, renal and/or thyroid disease, even when these diseases were adequately treated and/or controlled preoperatively (*p* = 0.0034, Objective Risk (*OR*) = 2.60, 95% CI 1.38–4.89). The likelihood of developing hyponatremia of any severity and due to any etiology was also significantly higher in female patients than in male patients (*p* = 0.011, *OR* = 2.18, 95% CI 1.20–3.96). No significant relationship was observed, however, between patient age (*p* = 0.83, *OR* = 1.0, 95% CI 0.98–1.015), the presence of preoperative anterior pituitary dysfunction (*p* = 0.68, *OR* = 0.88, 95% CI 0.47–1.64), preoperative tumor size (*p* = 0.64, *OR* = 1.006, 95% CI 0.98–1.03) or the extent of operative resection (*p* = 0.87, *OR* = 1.0, 85% CI 0.97–1.03) and the likelihood of developing postoperative hyponatremia.

### 3.9. Treatment of Hyponatremia

Thirty of the 62 patients with postoperative hyponatremia (48.4%) received no treatment. The majority of these patients had only mild hyponatremia, although three patients with moderate hyponatremia received no treatment. Treatment strategies in the other 32 hyponatremic patients included fluid restriction in 27 patients, diuresis (e.g., furosemide) in 19 patients, hypertonic saline in nine patients, normal saline in seven patients, sodium chloride tablets in three patients, demeclocycline in two patients, hydrocortisone and/or fludrocortisone in two patients and conivaptan in one patient. Hyponatremia treatments are summarized in Table 4.

### 3.10. Outcomes

Mean time to resolution of hyponatremia was 3.5 ± 4.1 days for all 62 patients, 2.0 ± 2.8 days for those with mild hyponatremia, 5.1 ± 5.1 days for those with moderate hyponatremia and 5.3 ± 3.6 days for those with severe hyponatremia. Time to resolution of hyponatremia was significantly greater for patients with moderate (*p* = 0.007) or severe (*p* = 0.003) hyponatremia when compared with patients having only mild hyponatremia. No significant difference between time to resolution of hyponatremia was seen amongst the different etiologies for hyponatremia (SIADH* vs.* CSW, *p* = 0.49; SIADH* vs.* desmopressin acetate, *p* = 0.43; CSW* vs.* desmopressin acetate, *p* = 0.27).

On univariate analysis, the use of fluid restriction in hyponatremia of any severity was a significant predictor of prolonged time to resolution of hyponatremia due to either CSW (*OR* = 5.14, *p* = 0.016) or SIADH (*OR* = 3.27, *p* = 0.008). Multiple regression analysis of treatment effect revealed that the use of diuretics was a significant, independent predictor of prolonged time to resolution of hyponatremia when used in SIADH of any severity (*OR* = 3.9, *p* = 0.02) or mild hyponatremia of any etiology (*OR* = 5.67, *p* = 0.009). Both the use of fluid restriction (*OR* = 2.48, *p* = 0.04) and the use of diuretics (*OR* = 3.15, *p* = 0.01) were also significant, independent predictors of prolonged time to the resolution of hyponatremia when used in hyponatremia of any severity or etiology. 

No treatment strategy significantly predicted decreased time to resolution of hyponatremia in univariate or multivariate logistic regression analysis. The use of hypertonic saline (*OR* = −2.4, *p* = 0.10), sodium chloride tablets (*OR* = −1.57, *p* = 0.45) and normal saline (*OR* = −0.44, *p* = 0.78) were the best predictors of the shortened time to resolution of hyponatremia of any etiology or severity, although the predictive value of these factors did not achieve statistical significance.

All patients with symptoms related to hyponatremia experienced full relief of symptoms after the resolution of hyponatremia. No deaths or persistent neurological deficits occurred as a result of hyponatremia. Outcomes of hyponatremia treatment are summarized in Table 4.

**Table 4:** Treatment strategies, treatment efficacy and clinical outcomes of postoperative hyponatremia seen in 62 patients after undergoing transsphenoidal resection of a pituitary mass at the authors’ institution between January, 2006, and December, 2012.

Hyponatremia Treatment Strategies (*N* (%))	
None	30 (48.4)
Fluid restriction	27 (43.5)
Diuretics (e.g., furosemide)	19 (30.6)
Hypertonic saline	9 (14.5)
Normal saline	7 (11.3)
Sodium chloride tablets	3 (4.8)
Demeclocycline	2 (3.2)
Corticosteroids	2 (3.2)
Conivaptan	1 (1.6)
**Time to Resolution of Hyponatremia (days)**	
All 62 patients with hyponatremia	3.5 ± 4.1
By Severity	
Mild hyponatremia (*N* = 32)	2.0 ± 2.8
Moderate hyponatremia (*N* = 19)	5.1 ± 5.1
Severe hyponatremia (*N* = 11)	5.3 ± 3.6
By Etiology	
Hyponatremia due to desmopressin acetate overdose (*N* = 3)	1.5 ± 0.9
Hyponatremia due to SIADH (*N* = 44)	3.7 ± 4.0
Hyponatremia due to CSW (*N* = 15)	4.3 ± 4.1
By Treatment	
Conivaptan (*N* = 1)	1
Normal saline (*N* = 7)	4 ± 2.4
Corticosteroids (*N* = 2)	4 ± 2.8
Sodium chloride tabs (*N* = 3)	4.7 ± 1.2
Hypertonic saline (*N* = 9)	4.8 ± 3.9
Fluid restriction (*N* = 27)	5.7 ± 4.8
Demeclocycline (*N* = 2)	6 ± 1.4
Diuretics (e.g., furosemide) (*N* = 19)	6.3 ± 5.1
**Clinical Outcomes of Hyponatremia (*N* (%))**	
Complete resolution of symptoms	62 (100)
Persistent neurological deficit	0 (0)
Death	0 (0)

## 4. Discussion

### 4.1. Etiology of Post-transsphenoidal Hyponatremia

Disorders of sodium balance are common after transsphenoidal surgery [19]. The mechanism underlying this predisposition is not entirely clear, but some authors have suggested that it may be attributable to the aberrant release of hormones (e.g., ADH from the neurohypophysis, cortisol from the adenohypophysis) or abnormal sympathetic hypothalamic outflow occurring as a result of surgical manipulation of the gland itself, the stalk or the hypothalamus [19].

Fisher [31] and others [32] described a now classic, triphasic trend of serum sodium abnormalities seen after transection of the infundibular stalk in both animals and humans (in which DI is initially seen, followed by hyponatremia related to SIADH several days later and a final phase consisting of DI, which often persists). Indeed, much of the early literature suggests that post-transsphenoidal hyponatremia (particularly when arising in a stereotypically delayed fashion, 5–7 days after surgery) is predominantly due to SIADH [33,34,35,36]. The classic triphasic response described by Fisher is only seen in 1%–2% of patients undergoing transsphenoidal surgery (1.6% in our series), however [19,37], and many patients with post-transsphenoidal hyponatremia demonstrate features (e.g., volume depletion) altogether inconsistent with SIADH. This discrepancy between clinical and experimental observation may be a manifestation of interventions (e.g., fluid restriction) administered in the clinical setting (but not in the experimental setting), as suggested by some authors [19], or alternatively, an illustration of this experimental model’s insufficiency in accurately characterizing the underlying pathology of post-transsphenoidal hyponatremia.

Olson* et al.* prospectively studied 92 patients undergoing transsphenoidal surgery, 25% of whom developed postoperative hyponatremia. Evidence of impaired suppression of ADH-secretion was seen not only in the hyponatremic patients, but also in two-thirds of the normonatremic patients. They demonstrated that the patients with impaired ADH suppression who failed to develop hyponatremia exhibited higher dietary sodium intake and lower natriuresis compared with hyponatremic patients, suggesting that the pathophysiology of post-transsphenoidal hyponatremia is complex and multifactorial and not due only to impaired ADH suppression, but instead displaying features of both SIADH and CSW [21].

Several reports of post-transsphenoidal hyponatremia in the literature suggest CSW as a primary etiology [38,39]. The pathophysiology of CSW is poorly understood, but CSW after transsphenoidal surgery has been suggested to arise as a result of the aberrant release of brain natriuretic peptide (BNP) and other natriuretic factors to the manipulation of the hypothalamic-pituitary axis, demonstrated in some studies to be related to an underlying abnormality of sympathetic outflow from the hypothalamus to the renal system, resulting in decreased renal tubular sodium resorption [40,41]. BNP [42,43], atrial natriuretic peptide (ANP) [44,45] and other natriuretic factors [46] have been suggested to serve as the molecular mediators of this abnormal natriuresis in CSW, although conclusive evidence of this relationship is lacking, and no identifiable consensus currently exists [4,29,40].

Secondary hypoadrenalism must also be considered in the differential diagnosis of post-transsphenoidal hyponatremia. Some authors suggest that the characteristically-delayed hyponatremia arising after transsphenoidal surgery is most often due to adrenocortical insufficiency [47,48]. As cortisol is known to inhibit vasopressin secretion [49,50], a role for hypoadrenalism in producing the SIADH-like effect seen in many patients with delayed post-transsphenoidal hyponatremia is not difficult to imagine. 

In our series, while the majority of post-operative hyponatremia was felt to be related to SIADH (71%), characteristics suggestive of CSW (e.g., hypovolemia, natriuresis) were seen in 24.2%. Secondary adrenal insufficiency was considered a contributing factor in 6.5%, over-administration of desmopressin acetate in 4.8% and excessive oral intake of hypotonic fluids in 1.6%.

Interestingly, patients in this series subjected to post-operative CSF diversion were at a significantly greater risk of hyponatremia development when compared with patients in whom CSF drainage did not occur (*p* = 0.0006), although neither the length (*R* = 0.023, *p* = 0.91) of CSF drainage nor the rate (*R* = 0.11, *p* = 0.59) of CSF drainage correlated with the severity of hyponatremia in these patients. Although the development of hyponatremia in direct relationship to CSF drainage has been reported only rarely in several case reports and small series [51,52,53,54], water retention in association with extrarenal sodium losses are a well-documented etiology of hypotonic, hypovolemic hyponatremia (e.g., as seen with excessive gastrointestinal losses) [55], and a similar mechanism is likely the underlying cause of hyponatremia development in these patients.

### 4.2. Incidence of Post-Transsphenoidal Hyponatremia

Though the reported incidences of post-transsphenoidal hypernatremia and hyponatremia vary considerably, results of most large, modern series indicate that hyponatremia is more common [17,18,19,20,21]. The incidence of post-transsphenoidal hyponatremia in this series (18%) is consistent with that seen in other large series (16%–38.8%) [19,20,21,56,57]. It has been suggested that the relatively stringent monitoring of electrolyte values seen in this series led to the early identification and treatment of hyponatremia, which may have otherwise developed into a delayed, more severe hyponatremia, thus falsely minimizing the incidence of delayed hyponatremia in this series. In this regard, it should be made clear that the majority of patients (17/27 patients, 63.0%) with early-onset (post-operative Day 0 or post-operative Day 1 onset) post-transsphenoidal hyponatremia in our series did not receive any treatment for their hyponatremia, and thus, it is felt unlikely that early treatment in these cases masked a delayed, more severe and/or more symptomatic hyponatremia. Furthermore, although data regarding the length of ICU stay and the stringency of laboratory monitoring trends after transsphenoidal surgery is relatively lacking in the literature, many large series report laboratory monitoring trends consistent with, or with greater stringency than, those described in this series [37,54,56,58].

Hyponatremia has been shown to lead to a considerable degree of morbidity and mortality in the literature [1,22,23,24]. Although no complications or persistent clinical consequences occurred in our series as a direct result of hyponatremia, the length of ICU stay (*OR* = 1.43, *p* ≤ 0.0005) and hospital stay (*OR* = 2.82, *p* < 0.0005) were both significantly higher in patients with hyponatremia.

In this series, a significantly higher incidence of post-operative hyponatremia was seen in female patients (*p* = 0.011, *OR* = 2.18) and in patients with cardiac, renal or thyroid disease (*p* = 0.0034, *OR* = 2.60), even when these disease were well-controlled. Other studies have shown a similar predisposition toward hyponatremia in female patients. The mechanism underlying this gender predilection is not entirely clear, but previous studies have documented a gender-specific difference in the antidiuretic response to antidiuretic hormone [59,60,61], a finding that may be particularly relevant after transsphenoidal surgery, as ADH suppression has been shown to be impaired in many post-transsphenoidal surgical patients [19,21]. Several prior series have documented an elevated risk of post-transsphenoidal hyponatremia in patients with macroadenomas [34], while others have shown an elevated risk for hyponatremia in microadenomas (proposed by some authors to be related to a greater degree of intraoperative exploration and hypophyseal manipulation occurring during microadenoma resection) [21]. This relationship between tumor size and hyponatremia risk was not seen in our series. Similarly, a large series by Jahangiri* et al.* documented an elevated risk for post-transsphenoidal hyponatremia in patients with preoperative hypopituitarism [56], but these findings were not reproduced in our series.

### 4.3. Onset and Clinical Manifestations of Post-Transsphenoidal Hyponatremia

According to the classically-described triphasic pattern of sodium imbalance after infundibular transection, polyuria and hypernatremia (due to diabetes insipidus) occur initially (2–3 days after transection) and are followed several days later (days 5–7 after transection) by oliguria and hyponatremia [31,32]. This timeline of events has also been observed clinically in some patients after transsphenoidal surgery and other neurosurgical procedures in or around the hypothalamic-hypophyseal system [36,62,63]. This so-called “delayed postoperative hyponatremia” is thought to arise due to the slow and delayed release of ADH stored within the magnocellular osmoregulatory neurons of the posterior lobe of the pituitary gland prior to the injury event [21,35,64,65], a theory bolstered in part by the experimental finding that this delayed hyponatremia can be avoided by preemptive removal of the neurohypophysis in canines [64].

The results of our study highlight an important caveat to the above principle, however, which is that the majority of hyponatremia (when defined as a serum sodium <135 mEq/L) actually occurs very early in the post-operative course (mean onset in our series: 3.2 ± 4.6 days postoperatively), is quantitatively mild, often asymptomatic and tends to resolve promptly and spontaneously. Though the mechanism underlying this association between early hyponatremia and mild hyponatremia is not entirely clear, some authors propose that this mild, early post-transsphenoidal hyponatremia is actually a different entity altogether and not related to ADH release, but rather to excessive administration of hypotonic fluids during surgery and in the immediate postoperative period [37,66]. The spontaneous resolution of this early hyponatremia could thus be explained as being due to renal clearance of this excess fluid volume, a function that occurs relatively rapidly in patients without renal disease. 

That the majority of patients with post-transsphenoidal hyponatremia lack identifiable clinical symptoms relatable to hyponatremia has been documented previously [19,20,21,51]. The clinical manifestations of hyponatremia often correlate well with the severity of hyponatremia (where mild hyponatremia is more often asymptomatic), as seen in this series.

### 4.4. Treatment of Post-Transsphenoidal Hyponatremia

The treatment for post-transsphenoidal hyponatremia should ideally be tailored to the underlying etiology in each case. The treatment for SIADH consists primarily of fluid restriction, as the prevailing pathophysiology in SIADH lies in inappropriate resorption of free water in the setting of normo or euvolemia, as a result of the influence of ADH on the late distal tubules and collecting ducts of the nephron [2,24,29]. The “gold standard” treatment for CSW, in contrast, is fluid resuscitation, although the most appropriate fluid type for replacement in CSW is a matter of debate [2,67,68]. Normal saline has traditionally been used, although early studies indicate that hypertonic saline is both safe [68] and effective [2,67].

Exogenous mineralocorticoids have also been used to treat hyponatremia in neurosurgical patients with some success, both in the setting of CSW [69,70] and of hyponatremia related to secondary adrenal insufficiency [2,29]. Early series examining the use of vasopressin receptor antagonists (e.g., conivaptan or tolvaptan) have also shown promising results, and though costly, these agents may be particularly useful in acute, severe hyponatremia, such as that seen in patients initially discharged and later readmitted with severe, symptomatic hyponatremia [56,65].

The use of hypertonic saline (*OR* = −2.4), sodium chloride tablets (*OR* = −1.57) and normal saline (*OR* = −0.44) were the most effective treatments in our series, regardless of hyponatremia etiology or severity, although the predictive value of these factors did not achieve statistical significance. Curiously, the use of fluid restriction tended to lengthen the required treatment in our series, even when used in patients with suspected SIADH. 

### 4.5. Study Weaknesses

The evaluation of hyponatremia in the neurosurgical patient, as in any patient or setting, should ideally progress systematically and according to a series of characteristic laboratory tests and clinical evaluations, all of which have been well-defined elsewhere [24,29]. In the clinical setting, however, certain steps in this evaluation can be overlooked and certain assumptions made, making the retrospective evaluation of hyponatremia etiology challenging. In several instances in our series, for example, a low serum osmolality was documented by laboratory analysis. In most cases, hyponatremia was presumed to be of the hypotonic variety, as this is the most common scenario, and no evidence existed to suggest otherwise (e.g., no patient was severely hyperglycemic, no patient was given mannitol and no history of administration of sodium-free irrigant solutes was present). Similarly, 24.2% of the hyponatremia in our series was felt to be related to CSW, although in most of these cases, urine sodium was not measured, and thus, the possibility that the hypovolemic hyponatremia observed in some of these patients was related instead to extrarenal solute loss (e.g., GI losses, skin losses) cannot be reliably ruled out. CSF diversion via a lumbar drain (seen in 22.7% of all patients, 40.3% of patients with hyponatremia of any etiology and 73.3% of hypovolemic hyponatremia, felt to be due to CSW) was likely to be a contributing factor, or perhaps the predominant underlying etiology, in a considerable portion of the patients with post-transsphenoidal hypovolemic hyponatremia in this series. If CSF diversion were theoretically considered to be the primary etiology of hyponatremia development in the 11 patients in this series with hypovolemic hyponatremia attributed to CSW, but in whom CSF diversion also occurred, then the incidence of hyponatremia due to CSW would decrease to 6.4% (from 24.2% as reported), and the incidence of hyponatremia due to extrarenal sodium loss would be 17.7%.

Furthermore, volume status, which plays a prominent role in the evaluation of hyponatremia, was assessed in this series by physical exam findings and other non-invasive measures. Assessing volume status by non-invasive measures, however, has been shown to be relatively unreliable, and doing so retrospectively may be particularly problematic. Neither volume status prior to surgery nor uric acid levels at the time of hyponatremia were routinely documented in this series. Additionally, although volume status was systematically monitored (through non-invasive monitoring of fluid intake and output) intraoperatively and post-operatively for patients in the ICU setting, volume status assessments were likely less reliable once patients were transferred to the regular, acute care setting.

Other weaknesses of this study include the tendency to use of a variety of different treatment methodologies for hyponatremia in the same patient (e.g., fluid restriction, hypertonic saline and NaCl tablets together), a practice that may not be unusual in the clinical setting, but one that makes it challenging to accurately evaluate the effectiveness of each treatment strategy in isolation. Furthermore, vasopressin receptor antagonists, which have been shown in some series to have a significantly greater efficacy in the treatment of post-transsphenoidal hyponatremia when compared with conventional therapies, were utilized in only a single patient in this series, preventing one from drawing reliable conclusions regarding their effectiveness in this patient population.

Somewhat surprising to the authors was the fact that no single treatment strategy used in this series was shown to significantly improve the rate of hyponatremia correction. In fact, many of the treatment strategies were found to negatively impact the rate of hyponatremia correction. This finding may be at least partially explained as a manifestation of selection bias, in that patients with more severe hyponatremia were more likely to receive one or more treatments for hyponatremia (compared to those with mild hyponatremia, who were more likely to go untreated), and thus, these treatment strategies appear to be associated with more severe hyponatremia and a slower rate of correction, whereas the converse statement may in fact be true. A randomized, prospective trial would be required to provide definitive evidence regarding the effectiveness of these and other treatment strategies for post-transsphenoidal hyponatremia.

## 5. Conclusions

The findings of this study and others indicate that hyponatremia is a relatively common finding after transsphenoidal surgery, and one with a complex pathophysiology. Rather than being attributable almost exclusively to SIADH, as is suggested by some authors, post-transsphenoidal hyponatremia is better described as both: (1) a conglomerate of multiple, distinct entities, each with an independent etiology and each tending to occur at characteristic time-intervals postoperatively; and (2) the collective influence of multiple, distinct etiologies working in concert to result in hyponatremia. Care should be taken to evaluate the etiology of hyponatremia in each case independently. Patients should be advised to monitor for signs and symptoms of hyponatremia after discharge, as hyponatremia may develop after discharge. Furthermore, treatment modalities should be tailored to the etiology of hyponatremia in each case, and the use of fluid restriction and diuretics should be de-emphasized in the treatment of post-transsphenoidal hyponatremia, as they have not been shown to significantly alter the time-course to the restoration of sodium balance. As the preferred treatment methodologies may differ drastically depending on the underlying etiology, misdiagnosis and improper treatment can have undesirable and, occasionally, fatal consequences. 
